# Reply to Borewad et al. Comment on “Rao et al. The Oncological Outcome of Postoperative Radiotherapy in Patients with Node-Negative Early-Stage (T1/T2/N0) Oral Squamous Cell Carcinoma and Perineural Invasion: A Meta-Analysis. *Cancers* 2025, *17*, 862”

**DOI:** 10.3390/cancers18111778

**Published:** 2026-05-29

**Authors:** Karthik N. Rao, Sreeram M. P., Remco de Bree, William M. Mendenhall, Primož Strojan, Göran Stenman, Antti Mäkitie, Alfons Nadal, Juan P. Rodrigo, Sweet Ping Ng, June Corry, Alessandra Rinaldo, Avraham Eisbruch, Alfio Ferlito

**Affiliations:** 1 Department of Head and Neck Oncology, Sri Shankara Cancer Foundation, Bangalore 560004, India; 2 Department of Head and Neck Surgical Oncology, Division of Imaging and Oncology Center, University Medical Center Utrecht, Heidelberglaan 100, 3584 CX Utrecht, The Netherlands; 3 Department of Radiation Oncology, University of Florida College of Medicine, Gainesville, FL 32610, USA; 4 Department of Radiation Oncology, Institute of Oncology Ljubljana, Faculty of Medicine, University of Ljubljana, 1000 Ljubljana, Slovenia; 5 Department of Pathology, Sahlgrenska Center for Cancer Research, University of Gothenburg, Sahlgrenska University Hospital, 41345 Gothenburg, Sweden; 6 Research Program in Systems Oncology, Department of Otorhinolaryngology-Head and Neck Surgery, University of Helsinki and Helsinki University Hospital, FI-00029 HUS Helsinki, Finland; antti.makitie@hus.fi; 7 Department of Pathology, Hospital Clinic, Barcelona, Department of Basic Clinical Practice, School of Medicine, Universitat de Barcelona, 08036 Barcelona, Spain; 8 Department of Otolaryngology, Hospital Universitario Central de Asturias, Instituto Universitario de Oncología del Principado de Asturias, University of Oviedo, Centro de Investigación Biomédica en Red de Cáncer (CIBERONC), 33004 Oviedo, Spain; 9 Department of Radiation Oncology, Austin Health, The University of Melbourne, Melbourne, VIC 3084, Australia; 10 Division of Radiation Oncology, GenesisCare Radiation Oncology, St. Vincent’s Hospital, Melbourne, VIC 3065, Australia; 11 ENT Unit, Policlinico Città di Udine, 33100 Udine, Italy; dottalerinaldo@gmail.com; 12 Department of Radiation Oncology, University of Michigan, Ann Arbor, MI 48109, USA; eisbruch@med.umich.edu; 13 Coordinator of the International Head and Neck Scientific Group, 35100 Padua, Italy; profalfioferlito@gmail.com

We sincerely thank Borewad et al. [1] for their constructive comments and for the time taken to engage deeply with our manuscript. We truly appreciate their careful appraisal and the opportunity it provides to further clarify our work [2].

The primary objective of our manuscript was to evaluate perineural invasion (PNI) in early oral cancer with pathologically node-negative disease, and to examine whether such patients derive a meaningful oncologic benefit from adjuvant postoperative radiotherapy (PORT). This clinically important question remains unresolved in routine practice, largely due to the limitations of the existing evidence base, as outlined in our meta-analysis [2].

As highlighted in our manuscript, we fully acknowledge that retrospective analyses are subject to heterogeneity in treatment practices across institutions and geographic regions, and this concern has been explicitly addressed in the limitations section of our manuscript.

With respect to the individual studies cited, Holcomb et al. presented a detailed subset analysis comparing PNI-positive and PNI-negative patients who received adjuvant PORT, and this relevant subset was appropriately included in our analysis [3]. Cheng et al. reported outcomes of PNI-positive and PNI-negative patients treated with PORT and chemotherapy separately; we included only the subset receiving adjuvant PORT alone and excluded those who received chemotherapy [4]. Similarly, Chen et al. provided separate subset analyses for PNI-positive and lymphovascular invasion-positive patients, from which we included only patients with PNI who received adjuvant PORT [5]. Nair et al. offered a detailed subset analysis of early oral cancers with node-negative, PNI-positive disease; accordingly, we included only patients fulfilling these criteria for our comparative evaluation [6].

Importantly, wherever additional adverse histopathological features were present in the parent cohorts of the included studies, only clearly defined subsets with isolated PNI were extracted for analysis, in keeping with the primary objective of this meta-analysis.

The data interpretation in our manuscript has been intentionally balanced. We have explicitly highlighted the limitations arising from variability in PNI nomenclature and reporting standards. As discussed, PNI is not a dichotomous variable, and its biological and prognostic significance is influenced by factors such as extent, nerve caliber, and frequent coexistence with other adverse histopathological features.

We are grateful for the identification of the numerical discrepancy. This is acknowledged as an inadvertent typographical error, and the correct total number is 529 rather than 522.

Finally, we wish to emphasize that this work reflects extensive collaborative effort, incorporating multiple rounds of review and the collective input of surgeons, radiation oncologists, and pathologists from diverse geographic regions, each contributing domain-specific expertise.

We sincerely value these comments, which not only clarify specific points of concern but also enhance the transparency and rigor of our work.

## Data Availability

No new data were generated in support of this research.
